# Inter-device reproducibility of transcutaneous bilirubin meters

**DOI:** 10.1038/s41390-020-01118-6

**Published:** 2020-09-12

**Authors:** Alida J. Dam-Vervloet, Marlijn D. van Erk, Nina Doorn, Stefan G. J. Lip, Nienke A. Timmermans, Leen Vanwinsen, Foky-Anna de Boer, Henrica L. M. van Straaten, Nienke Bosschaart

**Affiliations:** 1 Medical Physics Department, Isala hospital, Zwolle, The Netherlands; 2 Biomedical Photonic Imaging group, Technical Medical Centre, University of Twente, Enschede, The Netherlands; 3 Neonatology Department, Isala hospital, Zwolle, The Netherlands

## Abstract

**Background:**

Transcutaneous bilirubinometry is a widely used screening method for neonatal hyperbilirubinemia. Deviation of the transcutaneous bilirubin concentration (TcB) from the total serum bilirubin concentration (TSB) is often ascribed to biological variation between patients, but variations between TcB meters may also have a role. This study aims to provide a systematic evaluation of the inter-device reproducibility of TcB meters.

**Materials and Methods:**

Thirteen commercially available TcB meters (JM-105 and JM-103) were evaluated in vitro on phantoms that optically mimic neonatal skin. The mimicked TcB was varied within the clinical range (0.5–181.3 μmol/L).

**Results:**

Absolute differences between TcB meter outcomes increased with the measured TcB, from a difference of 5.0 μmol/L (TcB = 0.5 μmol/L phantom) up to 65.0 μmol/L (TcB = 181.3 μmol/L phantom).

**Conclusion:**

The inter-device reproducibility of the examined TcB meters is substantial and exceeds the specified accuracy of the device (±25.5 μmol/L), as well as the clinically used TcB safety margins (>50 µmol/L below phototherapy threshold). Healthcare providers should be well aware of this additional uncertainty in the TcB determination, especially when multiple TcB meters are employed in the same clinic. We strongly advise using a single TcB meter per patient to evaluate the TcB over time.

**Impact:**

- Key message: The inter-device reproducibility of TcB meters is substantial and exceeds the clinically used TcB safety margins.
- What this study adds to existing literature: The inter-device reproducibility of transcutaneous bilirubin (TcB) meters has not been reported in the existing literature. This in vitro study systematically evaluates this inter-device reproducibility.
- Impact: This study aids in a better interpretation of the measured TcB value from a patient and is of particular importance during patient monitoring when using multiple TcB meters within the same clinical department. We strongly advise using a single TcB meter per patient to evaluate the TcB over time.

## Introduction

Jaundice is a common and potentially harmful condition in neonates. Severe jaundice, or hyperbilirubinemia, may result in Kernicterus Spectrum Disorders (KSDs), causing irreversible brain damage to the patient.^1^ Therefore, screening of newborns at risk for hyperbilirubinemia is advised in international guidelines.^2,3^ Transcutaneous bilirubinometry is a widely used non-invasive and instantaneous method for this purpose. This method can reduce the number of invasive total serum bilirubin (TSB) determinations, which is considered as the golden standard.^4^ Transcutaneous bilirubin (TcB) measurements cannot completely replace TSB determinations, since the TcB concentration is a physiologically different parameter than the TSB.^5^

**Fig. 1:**
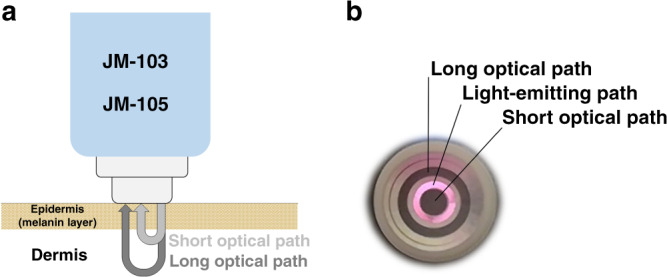
Design of the evaluated TcB meters (JM-103 and JM-105). **a** Schematic overview of the working principle of the evaluated TcB meters. Both types of meters have the same optical design. **b** Photograph of the tip of the evaluated TcB meter with light being emitted from the illumination ring (pink). The detection rings for the long and short optical path appear as dark gray in the photograph.

Transcutaneous bilirubinometry is based on optical spectroscopy and relates the optical absorption of bilirubin in (sub)cutaneous tissue to its concentration.^6^ Commonly used TcB meters, the Dräger JM-103 and JM-105, emit light with wavelengths of 450 nm (blue) and 550 nm (green).^7^ Both TcB meters correct for the background absorption of hemoglobin by employing the fact that bilirubin only absorbs light around 450 nm and hemoglobin absorbs at both wavelengths. Furthermore, their light collection geometry allows to distinguish between backscattered light which has traveled a short, and a long path (Fig. 1).^7^ The difference between both paths is used to correct for the influence of melanin absorption in the epidermal layer.^7^ Due to variations in optical illumination and detection geometry as well as analysis algorithms, other brands of TcB meters may perform differently.

Transcutaneous bilirubin meters use an internal calibration algorithm, based on the correlation between the TcB and the TSB, to convert the measured bilirubin absorption into an estimation of the TSB. Reported correlation coefficients between TcB and TSB determinations range from 0.39 to 0.95, resulting in accuracies of 4.2–45.5 µmol/L for TcB determinations.^8^ Clinically, this has the practical consequence that relatively large safety limits need to be employed (>50 µmol/L below the phototherapy threshold^2,4,9^) before additional TSB determinations can be omitted. One explanation for the variability in this correlation is the biological variation between and within patient populations: the TcB is a physiologically different parameter from the TSB and depends on the local extravasation of bilirubin into the skin.^5^ Biological variation may also give rise to differences in local skin anatomy that influence the probing volume of the TcB meter. In our recent work, we demonstrated that TcB determinations are influenced significantly (up to 72 µmol/L) by realistic variations in both bone depth and skin maturity related light scattering.^10^ Whereas biological variability is one explanation, another explanation for the variability in the correlation between the TcB and TSB may be the variability between TcB meters themselves. Until now, the inter-device reproducibility between TcB meters has remained unreported. A systematic evaluation of the inter-device reproducibility in vivo is practically impossible, due to inevitable physiological changes in the probed skin volume in between repeated TcB measurements, as well as difficulties in repositioning the TcB meter at exactly the same skin location. We, therefore, performed an in vitro study on the inter-device reproducibility of TcB meters. This aids in a better interpretation of the measured TcB value from a patient and is of particular importance for the purpose of patient TcB monitoring, when multiple TcB meters in one clinical department are in use.

## Materials and methods

### TcB meters

For this study, we evaluated thirteen TcB meters of the type JM-103 and JM-105 (Draeger Medical, Lübeck, Germany), which are clinically used in pediatric departments in hospitals across the Netherlands, see Table 1. Since the JM-105 is the successor of the JM-103, the user interfaces differ between both devices, but the optical design and measurement principles are identical. For both devices, the accuracy of the TcB measurements specified by the manufacturer is 25.5 μmol/L. All TcB meters were used under normal conditions and according to the instructions from the manufacturer. No information was available about the intensity of use of the TcB meters, but the date of installation was provided (Table 1).

**Table 1:** Included TcB meters, hospital, type, serial number, and date of installation.

TcB meter	Hospital	Type	Serial number	Date of installation
M1	Isala, Zwolle	JM-105	B3601086	9–2017
M2	Isala, Zwolle	JM-105	B3601137	7–2017
M3	Isala, Zwolle	JM-105	B3601107	10–2018
M4	Isala, Zwolle	JM-103	3201594	7–2013
M5	Isala, Zwolle	JM-105	B3601081	9–2017
M6	Medical Spectrum Twente, Enschede	JM-105	B3601005	10–2015
M7	Spaarne Hospital, Haarlem	JM-105	B3601050	4–2014
M8	Spaarne Hospital, Haarlem	JM-103	3201377	9–2017
M9	Spaarne Hospital, Haarlem	JM-103	3201007	1–2013
M10	University Medical Center Groningen, Groningen	JM-105	B3601390	3–2019
M11	University Medical Center Groningen, Groningen	JM-105	B3601402	3–2019
M12	University Medical Center Groningen, Groningen	JM-103	3202711	06–2011
M13	University Medical Center Groningen, Groningen	JM-103	3203135	01–2017

### Neonatal skin-mimicking phantoms

The inter-device reproducibility of the TcB meters was evaluated on six aqueous phantoms that accurately mimic the optical absorption and scattering properties of neonatal skin. Phantoms are generally used in medical diagnostics to evaluate device performance. Compared to the in vivo measurement situation on (neonatal) skin, the phantoms in this study are highly predictable, stable, and reproducible. This eliminates the uncertainties on sample instability that may arise from inevitable physiological tissue changes during the in vivo comparison of TcB meter performance.^11,12^ The phantoms were fabricated according to the procedure in our previous work,^10^ and the required optical properties were derived from an in vivo study on 60 neonates with varying gestational maturity.^13^

In brief, skin absorption by bilirubin and hemoglobin was mimicked by two dyes (Ecoline: Light-Yellow-201 and Magenta-337, Royal Thalens, The Netherlands). The mimicked TcB was varied by adapting the optical absorption around 450 nm through individual tuning of the concentrations of both dyes (Table 2). As the optical absorption around 450 nm scales linearly with TcB concentration, the higher the optical absorption of the phantom, the higher the mimicked TcB. The absorption at 550 nm was kept as constant as possible to mimic a stable cutaneous hemoglobin concentration of 2.13 g/L, which is the average value for neonatal skin.^13^ Optical scattering by neonatal skin is largely governed by skin maturity related collagen content^14^ and therefore varies substantially between newborns.^13^ For the phantoms of this study, light scattering was mimicked with dilutions of the standard tissue scattering phantom Intralipid (Intralipid® 20%, Fresenius Kabi, Bad Homburg, Germany) to a reduced scattering coefficient (*µ*_s_′) of 2.00 mm^−1^ at 450 nm and 1.63 mm^−1^ at 550 nm, which is the average value for neonatal skin.^13^

**Table 2:** Absorption properties of the neonatal skin-mimicking phantoms at 450 and 550 nm.

Phantom	*µ*_a_ 450 nm (mm^−1^)	*µ*_a_ 550 nm (mm^−^^1^)	Mimicked TcB^a^ (µmol/L)	Mimicked TcB^b^ (mg/dL)
F1	0.54	0.34	0.5	0.03
F2	0.97	0.34	55.8	3.2
F3	1.54	0.35	108.5	6.3
F4	2.13	0.35	143.4	8.3
F5	2.70	0.36	164.0	9.5
F6	3.28	0.36	181.3	10.5

Scattering properties were constant for all phantoms (*µ*_s_′ = 2.00 mm^−1^ at 450 and *µ*_s_′ = 1.63 mm^−1^ at 550 nm).

^a^Mimicked TcB is the average measure TcB over all meters.

^b^For those readers who are more familiar with bilirubin concentrations in mg/dL, we also display mimicked TcB in mg/dL. Conversion factor 1 µmol/L = 0.058 mg/dL bilirubin.

To prevent damage to the TcB meters, direct phantom contact was avoided by covering the measurement tip with a transparent, waterproof layer of a stretched Tegaderm^TM^ film dressing (1634W, 3M Healthcare, USA). The thickness of this film was measured by optical coherence tomography to be 44 ± 7 μm (mean ± standard deviation (SD)),^10,15^ which is comparable to the thickness of the neonatal epidermis.^16^

### Experimental set-up

The same experimental set-up was used as in our previous work,^10^ which will be briefly described next. The phantoms were covered by a rigid steel plate with an opening (Ø 8.8 mm) to accommodate the TcB meter (outer optical detection ring Ø 8.1 mm). This was to ensure that the measurement tip of the meter could be pressed down before each measurement, which is a requirement for the JM-103 and JM-105 to operate correctly. Direct contact between the Tegaderm^TM^ covered measurement tip and the phantom was ensured. Air bubbles that adhered to the phantom bottom were removed by gentle scraping with a rubber block and air bubbles that adhered to the steel plate were effectively removed by making contact with the phantom bottom, prior to the measurement. To minimize optical reflections, the steel plate was painted black.

### Method reproducibility

To adequately evaluate inter-device reproducibility, the reproducibility of the employed methods must be well known. We evaluated three factors that influence method reproducibility: (1) intra-device reproducibility, (2) phantom reproducibility, and (3) the reproducibility of covering the measurement tip of the TcB meter with Tegaderm^TM^ film. Table 3 gives an overview of the evaluation of the reproducibility of the employed methods. In general, the TcB was measured repeatedly for 21 times per phantom to obtain a good estimate of the spread in our data. Since only the absorption coefficient (*µ*_a_) per phantom is exactly known and the measured TcB may vary per meter, the average measured TcB over all TcB meters was used as a measure for the resulting TcB value per phantom.

**Table 3:** Evaluation of the reproducibility of the employed methods; the intra- and inter-device reproducibility and the maximal intra- and inter-device difference.

	Meter (Table 1)	Phantom (Table 2)	Number of Tegaderm^TM^ films	Number of measurements	Average TcB value (μmol/L)	Method reproducibility^a^ (μmol/L)	Maximal intra-device difference^b^ (μmol/L)	Inter-device reproducibility^c^ (μmol/L)	Maximal inter-device difference^d^ (μmol/L)
						Intra-device reproducibility		Inter-device reproducibility	
TcB device reproducibility	M1–M13	F1–F6	1 per meter	1638^f^	0.5 (F1) 55.8 (F2) 108.5 (F3) 143.4 (F4) 164.0 (F5) 181.3 (F6)	0.9 (M1) 0.9 (M2) 0.8 (M3) 1.6 (M4) 1.8 (M5) 0.8 (M6) 0.3 (M7) 1.6 (M8) 1.1 (M9) 0.7 (M10) 0.6 (M11) 0.8 (M12) 0.7 (M13)	5.0 (M1) 8.0 (M2) 6.0 (M3) 9.0 (M4) 11.0 (M5) 5.0 (M6) 1.0 (M7) 10.0 (M8) 5.0 (M9) 5.0 (M10) 3.0 (M11) 6.0 (M12) 5.0 (M13)	1.2 (F1) 9.8 (F2) 11.2 (F3) 14.6 (F4) 15.4 (F5) 18.8 (F6)	5.0 (F1) 33.0 (F2) 42.0 (F3) 57.0 (F4) 59.0 (F5) 65.0 (F6)
Phantom reproducibility^e^	M6	F3 (8×)	1	168^g^	109.4	0.5 (M6)	6.0 (M6)	n.a.	n.a.
Tegaderm^TM^ film reproducibility	M6	F3	5	105^h^	104.4	0.7 (M6)	7.0 (M6)	n.a.	n.a.

*n.a.* Does not apply.

^a^Intra-device reproducibility is quantified as the SD of all measurements per TcB meter and phantom, averaged over all phantoms.

^b^Maximal intra-device difference is quantified as the maximal difference between the highest and lowest TcB measurement per TcB meter, across all phantoms.

^c^Inter-device reproducibility is quantified as the SD of all TcB measurements of per phantom, across all TcB meters.

^d^Maximal inter-device difference is quantified as the difference between the highest and lowest TcB measurement of all TcB measurements per phantom, across all TcB meters.

^e^In order to evaluate phantom reproducibility we prepared phantom F3 eight times.

^f^21 measurements per phantom were performed on 6 phantoms and with 13 TcB meters resulting in a total of 21 × 6 × 13 = 1638 phantom measurements.

^g^21 measurements on phantom F3 (prepared 8×) were performed with TcB meter M6 resulting in a total of 21 × 8 = 168 phantom measurements.

^h^21 measurements on phantom F3 were performed with TcB meter M6 and with 5 Tegaderm^TM^ films resulting in a total of 21 × 5 = 105 phantom measurements.

Intra-device reproducibility was evaluated individually for the thirteen TcB meters in this study as the standard deviation (SD) of all TcB measurements per meter and phantom, averaged over all phantoms. The maximal intra-device difference was quantified as the maximal difference between the highest and lowest TcB measurement per TcB meter and phantom, across all phantoms.

Phantom reproducibility was evaluated through the preparation of eight identical phantoms with identical preparation procedures (8× F3, Table 2). A single TcB meter (M6, Table 1) was used to evaluate the TcB of these phantoms. We quantified phantom reproducibility as the average SD of all TcB measurements, across all eight phantoms.

The reproducibility of covering the measurement tip of the TcB meter with Tegaderm^TM^ film was evaluated on a single phantom (F3, Table 2), by covering one TcB meter (M6, Table 1) repeatedly with five different Tegaderm^TM^ films. The corresponding reproducibility was quantified as the average SD of all TcB measurements, across all five Tegaderm^TM^ films.

### Inter-device reproducibility of TcB meters

Inter-device reproducibility was quantified as the SD of all TcB measurements per phantom, across all TcB meters. The maximal inter-device difference was quantified as the maximal difference between the highest and lowest TcB measurement per phantom, across all TcB meters.

## Results

### Method reproducibility

The reproducibility of the employed methods was maximally 1.8 µmol/L (M5) for the intra-device reproducibility, 0.5 µmol/L for the phantom reproducibility, and 0.7 μmol/L for the reproducibility of covering the measurement tip of the TcB meter with Tegaderm^TM^ film (Table 3). The maximal intra-device difference was 11.0 µmol/L (M5).

### Inter-device reproducibility of TcB meters

**Fig. 2:**
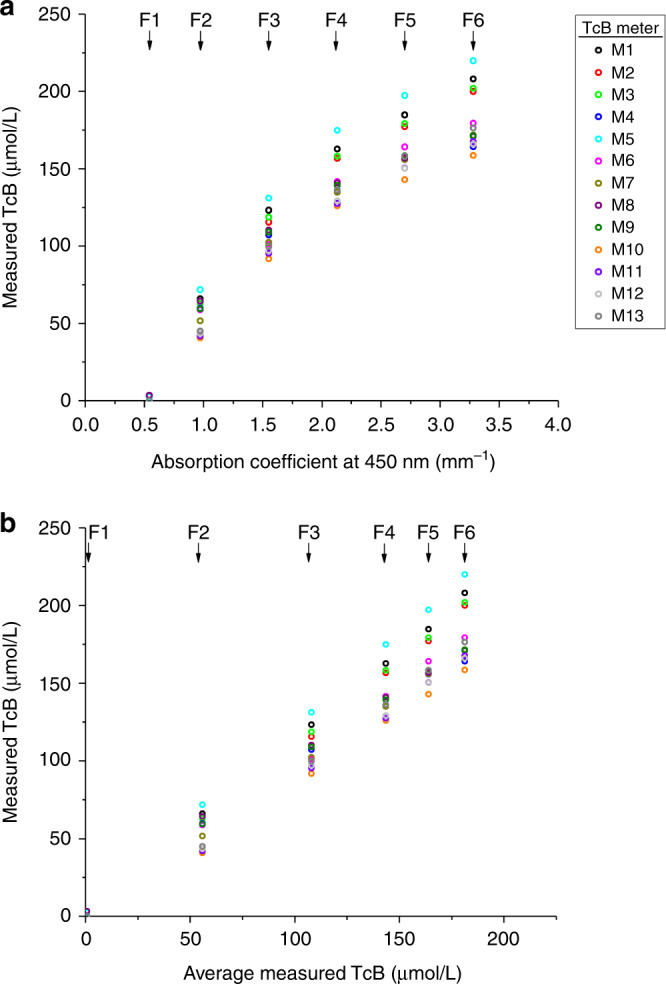
Inter-device reproducibility of TcB meters. Measured TcB values per TcB meter (M1–M13, Table 1) and phantom (F1–F6, Table 2) as a function of **a** the absorption coefficient of the phantom at 450 nm, and **b** the average measured TcB over all meters. Error bars (standard deviation of the TcB measurements per meter) fall behind the data points.

Figure 2 shows the measured TcB for all phantoms and TcB meters as a function of both the absorption coefficient at 450 nm (Fig. 2a) and the average measured TcB over all meters and phantoms (Fig. 2b). Adequate and reproducible performance of the TcB meters would ideally result in exactly the same TcB value for each phantom across all TcB meters. However, we observe a large dependency of the measured TcB value on TcB meter. Our data demonstrate that the variation between TcB meters increases with increasing TcB. The maximal difference in the measured TcB values amounts up to 65.0 μmol/L between meters M5 and M10 for phantom F6, at a mimicked TcB of 181.3 μmol/L.

These results, as well as the results for the inter-device reproducibility of the TcB meters, are listed in Table 3.

### Influence of TcB meter age

**Fig. 3:**
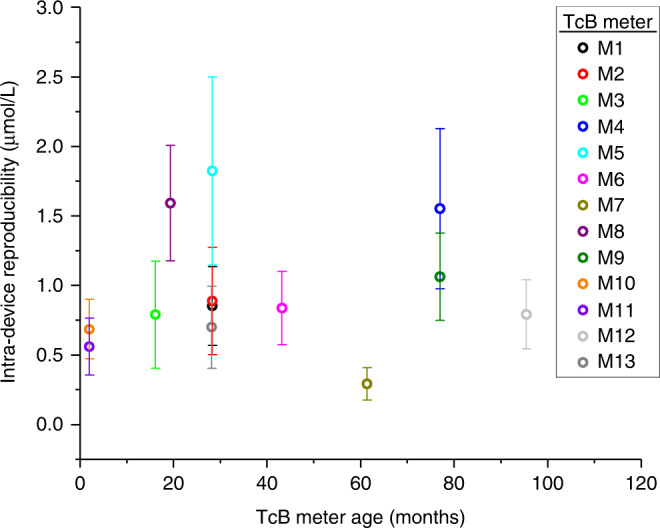
Influence of TcB meter age. Intra-device reproducibility (average SD over all measurements and phantoms per TcB meter) as a function of time since installation in the clinic (i.e., TcB meter age). The correlation coefficient between both parameters is *R* = 0.03. Error bars represent the standard deviation of the average SD across phantoms.

Figure 3 shows the relation between the time since installation in the clinic of the TcB meters (i.e., TcB meter age) and the average SD over all measurements and phantoms per meter. No correlation was found between this averaged SD and the age of the TcB meter (*R* = 0.03). Also, no relationship was found between the time since the last calibration by the manufacturer and the intra-device reproducibility of the TcB meters.

## Discussion

The main purpose of this study was to evaluate the inter-device reproducibility of TcB meters. Hereto, we evaluated thirteen TcB meters on neonatal skin-mimicking phantoms, in which we varied the mimicked TcB within the clinical range that naturally occurs in (premature) newborns. The accuracy of the TcB meter that has been specified by the manufacturer is 25.5 µmol/L for patients with a gestational age above 35 weeks, and 27.4 µmol/L for patients with a gestational age of 24 to 34 weeks.^7^ In this study, the maximum encountered difference between two different TcB meters in the measured TcB value on the same phantom was 65.0 μmol/L. This greatly exceeds the specified accuracy by the manufacturer.

The reproducibility of the employed methods within this study was good, with a maximal observed SD of 1.8 µmol/L (intra-device reproducibility). As we evaluated intra-device reproducibility on highly stable neonatal skin-mimicking phantoms, this number is substantially lower compared to the variation between repeated TcB measurements on patients reported in in vivo studies.^11^ The good reproducibility of our methods contributes to the reliability of the presented results on inter-device reproducibility. The maximum encountered difference in the measured TcB value on a single phantom with a single TcB meter and a single Tegaderm film was 11.0 µmol/L. Important to note here is that these variations may not only be due to method reproducibility but may also be ascribed to inconsistencies in the performance of the device itself. The large variation in the measured TcB values between TcB meters may (partly) be explained by the fact that the specified accuracy by the manufacturer was evaluated with a single TcB meter on a relatively homogeneous patient population. Figure 2 shows that the variation between TcB meters is caused by a structural offset, rather than a random variation in the measured TcB values per meter. For instance, TcB meter M5 structurally measures the highest TcB values on all phantoms. Contributing factors to these structural offsets may be small differences between meters in their individual calibration, as well as the geometry and efficiency of both the illumination and detection part of the TcB meter. The latter can be a result of the TcB meter manufacturing process, but it may also be affected by the degree of damage due to the intensity of TcB meter use. Since no information was available on the intensity of TcB meter use or signs of wear for this study, we investigated the correlation between intra-device reproducibility and the time since the TcB meter was installed in the clinic. No correlation was found between both parameters, therefore future studies are needed to unravel the exact causes of the large variations between TcB meters. Knowledge of these underlying causes will be of paramount importance for the improvement of inter-device reproducibility.

### Study limitations

Since a systematic in vivo evaluation of the inter-device reproducibility of TcB meters is practically impossible, we performed an in vitro study. We made use of highly predictable, stable, and reproducible neonatal mimicking skin phantoms, which accurately mimic the average optical properties of neonatal skin that have been previously assessed in vivo at the wavelengths detected by the TcB meter (450 and 550 nm).^13^ As demonstrated in our previous study on TcB performance,^10^ clinically realistic changes in these optical properties can directly influence the measured TcB. The inter-device reproducibility may therefore be different for non-average neonatal skin phantoms, which was not investigated in this study. We mimicked the TcB concentration up to a value of 181 µmol/L, which is above the phototherapy threshold for certain postnatal ages and risk groups.^2^ For TcB values >181 µmol/L, it is likely that the variability in measurement outcomes between devices will further increase (Fig. 2).

Although the reproducibility of the employed methods is high (≤ 1.8 μmol/L), our experimental set-up differs from the in vivo situation because the TcB meter was supported by a thin plate and we measured through a transparent Tegaderm^TM^ film dressing. The Tegaderm^TM^ film dressing can be considered part of the skin-mimicking phantom, as its thickness approaches that of the neonatal epidermis.^10^ For the employed measurement geometry, we assume that the thin plate does not contribute to the detection of any significant optical reflections. We did not mimic the epidermal melanin content in this study, therefore the mimicked phantoms are the only representative of Caucasian neonatal skin. Future phantom studies will also allow evaluating the inter-device reproducibility between different brands of TcB meters in a controlled manner.

### Clinical implications

This study provides insight into the inter-device reproducibility of transcutaneous bilirubin meters. With deviations up to 65.0 μmol/L between TcB meters, especially among the clinically relevant high TcB values, our results demonstrate that the clinically accepted safety margin (50 μmol/L below the phototherapy threshold) can be exceeded due to these device differences. Patient studies described in the literature report different correlation coefficients and variations between the TcB and TSB per patient, ranging from 0.39 to 0.95, and 4.2 to 45.5 µmol/L, respectively.^8^ Based on our observations, the lower correlations and high variations can be partly explained by the use of multiple TcB meters in one study. For future studies that evaluate TcB meter performance, a practical solution to avoid this uncertainty is to use only one TcB meter per patient. This will assure that the correlation between the TcB and the TSB remains unaffected by low inter-device reproducibility. However, the possibility of an absolute difference in measurement outcome between TcB meters should never be overlooked.

From a clinical perspective, this study aids to the clinical interpretation of the measured TcB value from an individual patient. Healthcare providers should be well aware that the decision to do an additional TSB determination can be influenced by the TcB meter in use. Our study is of particular importance during patient monitoring when using multiple TcB meters within the same clinical department. For follow-up patient measurements, this can lead to the under or overestimation of the progression of jaundice. Based on the results of this study, we strongly advise using a single TcB meter per patient to evaluate the TcB over time.

## Conclusions

In this study, we demonstrated that TcB determinations depend on the TcB meter itself, irrespective of the age of the device. Deviations up to 65.0 μmol/L between TcB meters of the same type greatly exceed the accuracy specified by the manufacturer (25.5 μmol/L). Healthcare providers should be aware of this additional uncertainty in the TcB determination and its consequences for clinical decision making. Based on the results of this study, we strongly advise using a single TcB meter per patient to evaluate the TcB over time.
